# A case of primary pulmonary leiomyosarcoma completely resected after neoadjuvant chemotherapy

**DOI:** 10.1186/s40792-019-0649-y

**Published:** 2019-05-31

**Authors:** Kazuhisa Tanaka, Takekazu Iwata, Kai Nishii, Yukiko Matsui, Tsukasa Yonemoto, Hidetada Kawana, Makiko Itami, Shigetoshi Yoshida, Toshihiko Iizasa

**Affiliations:** 1 Division of Thoracic Surgery, Chiba Cancer Center, 666-2, Nitona-cho, Chuo-ku, Chiba, 260-8717 Japan; 2 Division of Orthopedic Surgery, Chiba Cancer Center, Chiba, Japan; 3 Division of Surgical Pathology, Chiba Cancer Center, Chiba, Japan; 4 Department of Thoracic Surgery, International University of Health and Welfare School of Medicine, Narita, Japan

**Keywords:** Primary pulmonary leiomyosarcoma, Surgery, Neoadjuvant chemotherapy

## Abstract

**Background:**

Primary pulmonary leiomyosarcoma is a rare malignant tumor. We herein report a case of primary pulmonary leiomyosarcoma that was completely resected by surgery after neoadjuvant chemotherapy.

**Case presentation:**

A 60-year-old man presented with cough. Chest computed tomography showed an 11-cm mass in the right upper lobe of the lung that had invaded the superior vena cava. Endobronchial ultrasound-guided transbronchial needle aspiration revealed leiomyosarcoma of the lung. We considered complete resection of the tumor to be very difficult because of the tumor invasion into the right atrium inflow of the superior vena cava, so we performed chemotherapy using doxorubicin for five cycles. After chemotherapy, the tumor size decreased to 5.6 cm, and we performed right upper lobectomy with combined resection of the superior vena cava. The tumor was completely resected by surgery. The patient is alive without recurrence 17 months postoperatively.

**Conclusions:**

We encountered a case of primary pulmonary leiomyosarcoma that was successfully treated by surgery after neoadjuvant chemotherapy. Doxorubicin monotherapy was effective in this case. Surgery combined with neoadjuvant chemotherapy should be considered for such cases, as a long-term survival can be achieved by complete resection of primary pulmonary leiomyosarcoma.

## Background

Primary pulmonary leiomyosarcoma is a rare malignant tumor arising from the smooth muscle of the pulmonary parenchyma, pulmonary arteries, and the bronchial tree. The incidence has been reported to be less than 0.5% of all malignant pulmonary tumors and 30% of primary sarcomas of the lung [1–3]. We herein report a case of primary pulmonary leiomyosarcoma that was successfully resected by surgery after neoadjuvant chemotherapy.

## Case presentation

**Fig. 1:**
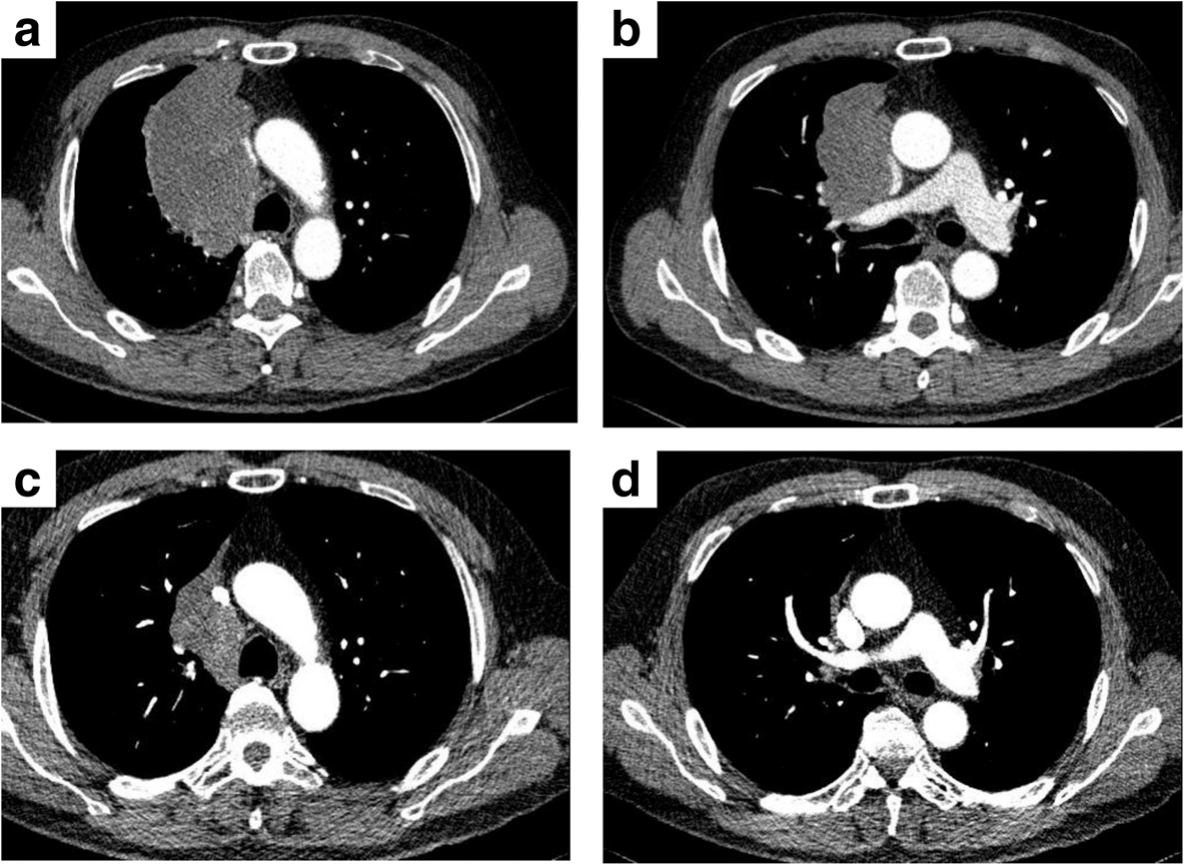
**a**, **b** Chest computed tomography (CT) before treatment showed an 11.0-cm mass in the right upper lobe that had invaded the superior vena cava (from the right internal jugular vein to the right atrium). **c**, **d** CT after chemotherapy showed a 5.6-cm mass in the right upper lobe. The reduction rate of the tumor size was 49.1%

**Fig. 2:**
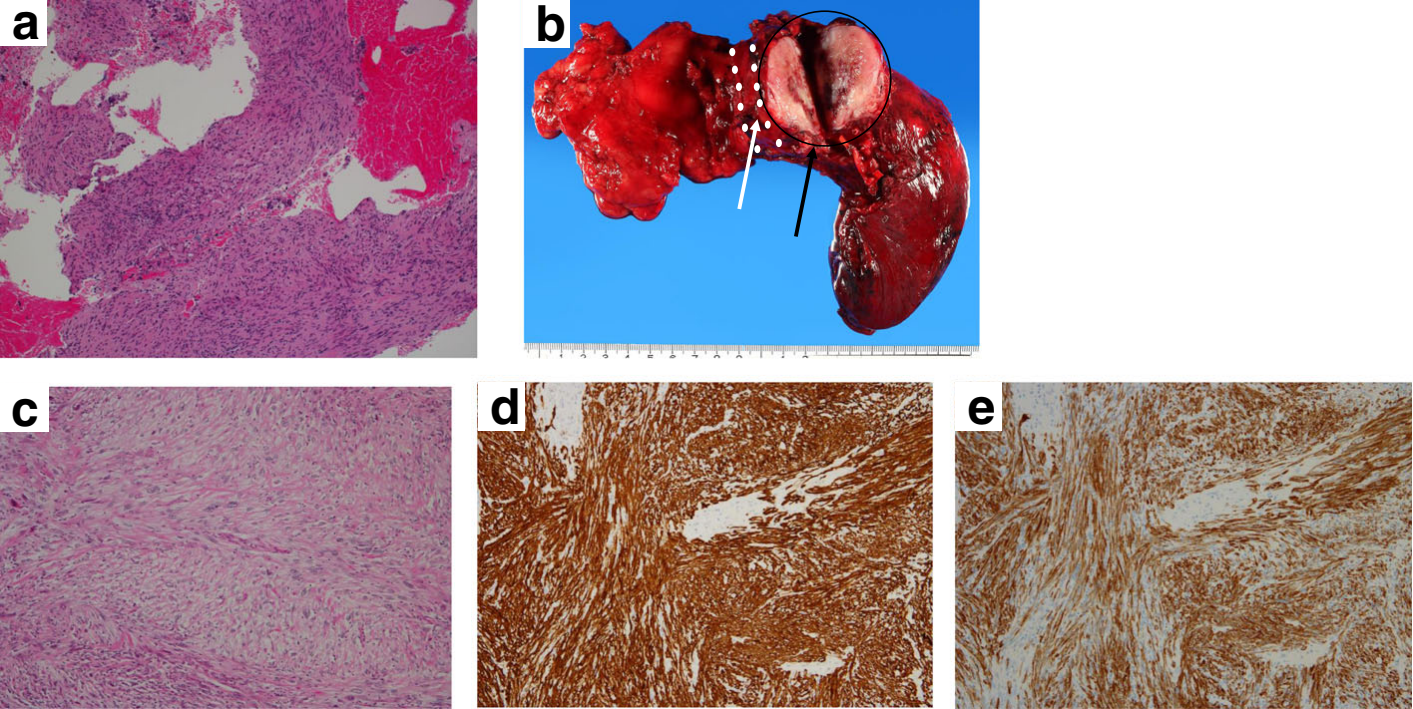
**a** EBUS-TBNA samples showed densely packed spindle-shaped tumor cells arranged in a fascicular pattern (H&E staining, × 100). **b** Macroscopic findings showed a yellowish tan tumor (8.0 × 4.8 × 4.0 cm) in the right upper lobe that had invaded the superior vena cava. The white arrow indicates the superior vena cava. The black arrow indicates the tumor. **c** Densely packed spindle tumor cells arranged in a fascicular pattern. The mitotic count was 16 mitoses per 10 high-powered field (H&E staining, × 100). **d** Immunohistochemical staining of the spindle tumor cells showed positive findings for smooth muscle actin (× 100). **e** Immunohistochemical staining of the spindle tumor cells showed positive findings for h-caldesmon (× 100)

A 60-year-old man was admitted with cough. Chest computed tomography (CT) showed an 11-cm mass in the right upper lobe that was suspected of invading the right side of the superior vena cava (SVC) almost from the proximal end of the right internal jugular vein to the right atrium inflow (Fig. 1a, b). The patient did not present with signs or symptoms of SVC obstruction. Endobronchial ultrasound-guided transbronchial needle aspiration (EBUS-TBNA) showed leiomyosarcoma of the lung (Fig. 2a). ^18^F-fluorodeoxyglucose (FDG) positron emission tomography (PET)-CT showed the high accumulation of FDG in the pulmonary tumor with a maximal standardized uptake value of 16.83; the absence of any other accumulation allowed us to exclude metastasis from another site. We diagnosed the patient with primary pulmonary leiomyosarcoma.

We considered complete resection of the tumor to be very difficult because of the tumor invasion, particularly that to the right atrium inflow of the superior vena cava, so we performed chemotherapy (doxorubicin monotherapy) for five cycles. We discussed the regimen for neoadjuvant treatment in a conference with orthopedic surgeons and medical oncologists. The recommended regimen for advanced soft tissue sarcoma is doxorubicin monotherapy or the combination of doxorubicin and ifosfamide. However, given that the combination therapy results in bone marrow toxicity more frequently than monotherapy, we selected doxorubicin monotherapy for neoadjuvant treatment. After chemotherapy, the tumor size shrank to 5.6 cm, and the distance of suspected invasion to the superior vena cava was reduced, rendering the tumor resectable with a sufficient surgical margin between the inflow of the atrium and the tumor (Fig. 1c, d).

We performed right upper lobectomy with combined resection of the superior vena cava and reconstructed the blood flow by an artificial vascular graft between the left brachiocephalic vein and right atrial appendage. We were able to remove the dense adhesion around the right brachiocephalic vein and right atrium inflow of the superior vena cava. However, we had to resect the superior vena cava around the azygous vein combinedly. The pathological findings revealed tumor invasion to the superior vena cava at that point. The tumor was completely resected by surgery (Fig. 2b). Pathological findings showed leiomyosarcoma and positivity for smooth muscle actin (SMA) and h-caldesmon (Fig. 2c–e). The tumor was found to have arisen in the lung and invaded the superior vena cava. The vessel lumen of the superior vena cava was intact, and the tumor had invaded the superior vena cava from the outside. The pathological response to neoadjuvant chemotherapy was Ef.1a. The patient was still alive without recurrence at 17 months postoperatively.

## Discussion

Most commonly, pulmonary sarcomas are of metastatic origin. Consequently, it is important to exclude metastasis from other sites, such as the gastrointestinal tract, uterus, or soft tissue, before establishing a diagnosis of primary pulmonary leiomyosarcoma [1, 2]. Most primary sarcomas of the lung occur in middle age and are more prevalent among men than women [1–3]. Primary pulmonary leiomyosarcoma causes unspecific symptoms, including chest pain, dyspnea, a fever, and cough, but it can also be asymptomatic. Thoracic symptoms are similar to those seen in epithelial tumors and depend on the location of the tumor [1–4]. Therefore, the early detection of primary pulmonary leiomyosarcoma is difficult. The reported radiographic findings of primary pulmonary leiomyosarcoma are a round, well-outlined mass [5]. The location of tumors is most commonly in the upper lobes [1].

Bao-Dong et al. conducted a population cohort study of 231 primary pulmonary leiomyosarcoma patients. They found that surgical resection and distant stage were independent prognostic factors for primary pulmonary leiomyosarcoma, and complete resection decreased risk of death by 57%. They also showed that the median overall survival (OS) for this disease was 14.0 months, and the 1-, 3-, and 5-year OS values were 52.7%, 29.0%, and 22.2% [3]. Effective chemotherapy is unavailable for most sarcomas. Anthracycline-based regimens using doxorubicin as the main chemotherapeutic agent were used in early studies, while more recent trials have tested doxorubicin combined with ifosfamide [6–8]. A randomized trial showed that neoadjuvant epirubicin and ifosfamide improve the survival of patients affected by five high-risk soft tissue sarcoma histologies of the trunk and extremities, including undifferentiated pleomorphic sarcoma, myxoid liposarcoma, synovial sarcoma, malignant peripheral nerve sheath tumors, and leiomyosarcoma [9]. The advantage of neoadjuvant chemotherapy is the achievement of negative histologic margins and improved chances to undergo conservative surgery. There have been no reports about neoadjuvant chemotherapy for primary pulmonary leiomyosarcoma. In our case, neoadjuvant chemotherapy using doxorubicin was effective. The tumor was considered unresectable before chemotherapy, but we were ultimately able to resect it completely.

## Conclusion

In summary, we encountered a case of primary pulmonary leiomyosarcoma that was successfully treated by surgery after neoadjuvant chemotherapy. Doxorubicin monotherapy was effective in this case. Surgery combined with neoadjuvant chemotherapy should be considered for such cases, as a long-term survival can be achieved by complete resection of primary pulmonary leiomyosarcoma.

## Data Availability

Not applicable.

## Abbreviations

CT: Computed tomography
EBUS-TBNA: Endobronchial ultrasound-guided transbronchial needle aspiration
FDG-PET: ^18^F-fluorodeoxyglucose positron emission tomography
OS: Overall survival
SVC: Superior vena cava

## References

[1] Etienne-Mastroianni B, Falchero L, Chalabreysse L, Loire R, Ranchere D, Souquet PJ, Cordier JF (2002). Primary sarcomas of the lung: a clinicopathologic study of 12 cases. Lung Cancer..

[2] Litzky LA (2008). Pulmonary sarcomatous tumors. Arch Pathol Lab Med..

[3] Qin BD, Jiao XD, Zang YS (2018). Primary pulmonary leiomyosarcoma: a population-based study. Lung Cancer..

[4] Elouazzani H, Zouaidia F, Jahid A, Bemoussi Z, Mahassini N (2012). Primary endobronchial leiomyosarcoma of the lung: clinical, gross and microscopic findings of two cases. J Clin Imaging Sci..

[5] Fitoz S, Atasoy C, Kizikaya E, Basekim C, Karsli F (2000). Radiologic findings in primary pulmonary leiomyosarcoma. J Thorac Imaging..

[6] Akin S, Dizdar O, Karakas Y, Turker A, Kars A (2018). Ifosfamide and doxorubicin in the treatment of advanced leimyosarcoma. Curr Probl Cancer..

[7] Judson I, Verweij J, Gelderblom H, Hartmann JT, Schoffski P, Blay JY, Kerst JM, Sufliarsky J, Whelan J, Hoheberger P, Krarup-Hansen A, Alcindor T, Marreaud S, Litiere S, Hermans C, Fisher C, Hogendoorn PC, dei Tos AP, van der Graaf WT (2014). European Organisation and Treatment of Cancer Soft Tissue and Bone Sarcoma Group. Doxorubicin alone versus intesified doxorubicin plus ifosfamide for first-line treatment of advanced or metastatic soft-tissue srcoma: a randomised controlled phase 3 trial. Lancet Oncol.

[8] Sleijfer S, Ouali M, van Glabbeke M, Krarup-Hansen A, Rodenhuis S, Le Cesne A, Hogendoorn PC, Verweij J, Blay JY (2010). Prognostic and predictive factors for outcome to first-line ifosfamide-containing chemotherapy for adult patients with advanced soft tissue sarcomas: an exploratory, retrospective analysis on large series from the European Organization for Research and Treatment of Cancer-Soft Tissue and Bone Sarcoma Group (EORTC-STBSG). Eur J Cancer..

[9] Pasquali S, Gronchi A (2017). Neoadjuvant chemotherapy in soft tissue sarcomas: latest evidence and clinical implications. Ther Adv Med Oncol..

